# War-related quality of life is associated with depressive symptoms and hopelessness among Palestinians: sense of belonging and resilience as mediating variables

**DOI:** 10.1017/gmh.2022.52

**Published:** 2022-10-17

**Authors:** Fayez Mahamid, Guido Veronese, Dana Bdier

**Affiliations:** 1 An-Najah National University, Nablus, Palestine; 2 University of Milano-Bicocca, Milano, Italy

**Keywords:** Depressive symptoms, Palestine, resilience, sense of belonging, war-related quality of life

## Abstract

**Background:**

The current study was designed to test the correlation between quality of life, depressive symptoms, and hopelessness, and whether sense of belonging and resilience mediating the correlation between quality of life, depressive symptoms, and hopelessness in a society characterized by high level of political violence and prolonged trauma.

**Methods:**

Structural equation modeling (SEM) was performed to test the conceptual model, where quality of life was identified as a predictor variable, sense of belonging and resilience as mediating variables, and depressive symptoms and hopelessness as outcome variables. The participants of the study were 437 Palestinian adults: 190 males and 247 females, they were recruited using online methods; emails, Facebook, and Twitter.

**Findings:**

Results indicated that quality of life negatively correlated with depressive symptoms (*r* = −0.603; *p <* 0.01), and hopelessness (*r* = −0.453; *p <* 0.01), and positively correlated with resilience (*r* = 0.534; *p <* 0.05), and sense of belonging (*r* = 0.428; *p <* 0.01). Results of SEM indicated the correlation between quality of life, depressive symptoms, and hopelessness was fully mediated by the sense of belonging and resilience.

**Conclusions:**

Our study sheds light on resilience and sense of belonging as protective factors against ongoing traumatic experiences among Palestinians. Future research should be addressed to understand better the features of resilience and sense of belonging that can help maintain psychological functioning in conditions of chronic and ongoing violence, the personal and historical antecedents of such protective factors, and the factors that can directly or indirectly undermine them.

## Introduction

During the past 73 years, Palestinians have experienced several catastrophic and violent antecedents characterized by invasions, detentions, land confiscations, house evictions, and demolitions (Veronese *et al*., 2014, 2017a, 2017b). Moreover, Palestinian people have been exposed to different direct and indirect traumatic circumstances as a result of the political conflict, Palestinians also suffer from restriction of movements between regions as a result of checkpoints and wall, this wall has been defined by many Palestinians as the ‘Apartheid Wall’ and by Israeli state leaders as a ‘security fence’. The construction of the wall has resulted in newer and catastrophic burdens for the Palestinians (Shalhoub-Kevorkian, 2006; Thabet *et al*., 2014; Mahamid, 2020).

Regarding the political situation, Palestinians had to face different challenges on several bases such as the militarization of the territory, poverty, lack of employment opportunities, economic dependence, violation of justice and equality, territorial fragmentation, building restrictions, cultural pressures, future insecurity, fewer positive social outlets due to the restrictions on movement between communities, lack of recreational facilities, lack of health and mental health care services, and destruction of social networks (Barber *et al*., 2014; Berte *et al*., 2021; Mahamid and Veronese, 2021; Mahamid and Bdier, 2020a; Veronese *et al*., 2021b). These challenging living conditions are considered a significant risk factor for mental distress and Palestinians' poor quality of life (QoL) (Veronese *et al*., 2017a, 2017b).

Thus, the first focus of our study is to examine the relationship between QoL, depressive symptoms, and hopelessness among Palestinian adults, and whether a sense of belonging (SOB) and resilience mediate the relationship between QoL (predictor) and the remaining two variables (depression and hopelessness as outcome variables).While there is a wealth of data to support these research questions in Western societies, few studies regarding this phenomenon have been conducted in non-Western societies. No previous research was found to test the correlation between these variables in the Palestinian context.

## Theoretical background

### War-related quality of life

One of the best indicators of people's living conditions in Palestine refers to the construct of QoL, best defined as how individuals evaluate the ‘goodness’ of multiple aspects of their lives, in which these evaluations include one's subjective emotional reactions to life occurrences, disposition, sense of life fulfillment and satisfaction, and satisfaction with working life and personal relationships (Theofilou, 2013). In the Palestinian context, where violence and insecurity are pervasive in everyday lives, political freedom, self-determination, participation in democratic processes, and political decision-making are essential factors fostering people's QoL and wellness (Giacaman *et al*., 2007; Mahamid and Berte, 2020).

Over generations, the intractable and entrenched Israeli/Palestinian conflict resulted in an undermined QoL for the population living in the occupied Palestinian territory (oPt) (Mataria *et al*., 2009). For example, Harsha *et al*. (2016) found that about one-third of adult Palestinians reported low levels of well-being, while Mahamid *et al*. (2021) found that traumatic life events were negatively associated with psychological well-being among Palestinians living in West Bank. Moreover, low health-related QoL (physical, psychological, and environmental) was significantly associated with war-related factors, especially mental distress, insecurity, and psychological suffering among Palestinian adults (Abu-Rmeileh *et al*., 2012).

### Ware-related quality of life and depressive symptoms

The challenging living conditions that Palestinians are experiencing on a daily base are found to be associated with poor QoL which is considered as a risk factor for poor psychological functioning. Therefore, McNeely *et al*. (2014) found that human insecurity and chronic economic constraints were positively associated with depressive symptoms in oPt. Furthermore, prolonged exposure to violence was associated with high depression and suicidal ideation levels among Palestinian young adults (Hamdan and Hallaq, 2021). Besides, exposure to violence, such as personal victimization, witnessing others’ oppression, was associated with a high prevalence of depression among Palestinians in the West Bank and East Jerusalem (Wagner *et al*., 2020). In contrast, higher QoL emerged as an indicator of decent well-being, while traumatic events were positively associated with psychological dysfunction and depression among Palestinians in the Gaza Strip (Thabet, 2017).

### War-related quality of life and hopelessness

Hopelessness is one of the states of mind that characterizes individuals living in war-like conditions and exposed to severe hardships (Boffa *et al*., 2018; Amir, 2020). Palestinians emerged to be hopeless from living in normal conditions due to the ongoing political and military violence that disrupt their daily life (Rabaia *et al*., 2010). Hopelessness involves very negative feelings and expectancies toward oneself, severe pessimism, and is often shaped by adverse life events that result to be out of control and impossible to overcome from individuals who experience it (Afifi *et al*., 2013).

Accordingly, the Hopelessness Theory of Depression described hopelessness as the consequence of (a) a negative expectancy about the future and (b) a simultaneous inability to control those future outcomes (Abramson *et al*., 1989). Hence, Palestinians may perceive a sense of hopelessness and its negative correlates because of conditions of severe uncertainty, insecurity, and economic instability (Diab *et al*., 2018).

### Quality of life and sense of belonging

On the contrary, a SOB and its power to give sense to people's lives is considered a protective factor against mental distress and poor war-related QoL (Lambert *et al*., 2013). SOB can be defined as the individual's experience of personal involvement in a system that makes them feel valued and involved in the community life, or a sense of fit between the person and the system (Kim *et al*., 2010). Also, belonging is a sort of perception or evaluation of how one feels. Such evaluation could relate to the quality of social connections, meaning, satisfaction with social ties, or the way someone feels toward a significant place or a relevant historical event (Allen, 2020). SOB has been broken into four main dimensions: membership, influence, integration/fulfilment of needs, and shared emotional connections (Banat and Rimawi, 2017).

A greater SOB enhances individuals' mental health and coping skills in adjusting to unexpected life events or stressors, providing people with a sense of purpose, meaning, and worth (Smith, 2011). Palestinians were found to display high levels of SOB and sense of community in sharing values, norms, experiences, a shared destiny, and history before and under the Israeli military occupation (Banat, 2014). Moreover, Palestinians belong to a society that has succeeded in maintaining its coherence as a protective factor against psychological distress, low self-confidence, and trauma-related outcomes (Chatty, 2009; Veronese and Pepe, 2014).

### Quality of life and resilience

Resilience is expected to be another human quality that enhances QoL and promotes mental health among individuals experiencing ongoing conflict and violence (Bosqui and Marshoud, 2018). The construct of resilience encompasses several definitions, such as a personal trait characterizing individuals and a process connecting people with their living environment (Masten and Reed, 2002; Pan and Chan, 2007). In the present study, resilience has been considered the people's capability to achieve positive outcomes despite challenging or threatening circumstances, cope successfully with traumatic experiences, and use positive problem-solving strategies to deal with intense grief (Zolkoski and Bullock, 2012; Mahamid and Bdier, 2020b).

Whereas in the Palestinian culture, resilience might be conceptualized as a prerequisite to understanding and achieving the so-called state of ‘*sumud*’; meaning that the individuals can be resilient whenever they are capable of remaining steadfast in the face of daily challenges, not giving up their place or position (Marie *et al*., 2018). God and a sense of spirituality can sustain people so that things happen for a reason in the Palestinian and overall Muslim faith. God drives a meaning for what one is acting, and blesses people's actions promoting their health and resilience even in the wake of hardships and traumatic circumstances (Thabet, 2017).

Moreover, Palestinian people settled a culture of resilience that supported them in enhancing their psychological well-being and QoL. As demonstrated by a cross-national comparative study involving Palestinian and Jordanian adults, levels of well-being and QoL were more vital among Palestinians. In contrast, stress levels were lesser than in their Jordanian counterpart due to the Palestinian cultural resilience, despite Jordanians living in peace and relatively free (Asi *et al*., 2018). Finally, resilience emerged as positively associated with positive emotions and good psychological functioning among Palestinian adults (Kteily-Hawa *et al*., 2020).

### The current study

#### Setting

We conducted our study during the recent war on Gaza (May–June 2021) and simultaneous escalation of the military violence in the West Bank of oPt in May 2021. The current outbreak of fighting began with a series of controversial Israeli actions in Jerusalem, including the attempted eviction of Palestinian families in East Jerusalem by right-wing Jewish settlers, an Israeli police raid on Palestinian worshippers at al-Aqsa Mosque during the month of Ramadan, a series of planned provocative march by far-right Israelis in the Arab/Palestinian side of the city. The military assaults on Gaza have continued their back-and-forth strikes for days, resulting in hundreds of deaths and widespread property damage, primarily but not exclusively on the Palestinian side, and the massive destruction of Palestinian infrastructures in the Gaza Strip. Furthermore, hundreds of Palestinians in the West Bank were injured due to mass protests against the attacks on Gaza (United Nations Human Rights Council, 2021).

Based on prior research (Chatty, 2009; Rabaia *et al.*, 2010; Abu-Rmeileh *et al*., 2012; Veronese and Pepe, 2014; Harsha *et al*., 2016; Boffa *et al*., 2018; Amir, 2020; Kteily-Hawa *et al*., 2020; Mahamid *et al*., 2021), study hypotheses were defined: *First, quality of life would be associated negatively with depressive symptoms (H1); second, quality of life would be associated negatively with hopelessness (H2); third, sense of belonging and resilience would mediate the association between quality of life and depressive symptoms (H3).*

## Methodology

### Participants

Participants were recruited from online advertisements, email campaigns, and social media. The aims of this study, along with the procedures, were presented, and interested participants sent an email indicating their willingness to participate. Each participant then received a letter briefly explaining the subject of the study and its purpose, mentioning all ethical issues of confidentiality and voluntary participation. Upon reading and accepting the conditions outlined in the email, participants replied with their written informed consent. Participants were (437) Palestinian adults: (190) males and (247) females. In total, 46% of participants were from cities, 54% were from villages. In total, 20.8% of participants were aged 18–25, 31.8% of participants were aged 26–33, 41% of participants were aged 34–42, and the remainder 6% were aged 44–51. In total, 66.6% of participants hold a graduate degree, 31.8% of participants were with an undergraduate degree, and the remainder 1.6% were with a high school degree. Inclusion criteria in the study required participants to be Palestinian, native Arabic speakers and living in the West Bank of the oPt during the last war on Gaza and military violence in the West Bank of Palestine.

### Measures

Following standard methodological recommendations for the development of our questionnaires (Hambleton *et al*., 2005), items (PHQ-9; BHS; GBS; RSA) were translated and back-translated from the original English version to Arabic and pilot-tested by a panel of 10 Arab professionals recognized as experts in psychology, counseling, and social work. These professionals evaluated the clarity and relevance of the questions and translation. After completing the translated draft, the questionnaires were back-translated into English by an independent mother tongue English editor. The translated version was then pilot tested among 70 participants and further refined for clarity according to their comments.

#### The World Health Quality of Life Scale (WHOQOL-Bref) Arabic version

The original WHOQOL scale was built on 100 questions to assess individuals' satisfaction and well-being over six QoL domains. The WHOQOL-Brief is a summarized version (using 26 questions) of the original WHOQOL scale. This shortened instrument has been validated in various international field trials and thus is considered appropriate to measure the QoL of both ill and well populations. Additional field trials have shown that it is a valuable instrument in epidemiological studies comparing the impact of different conditions on health and QoL. The instrument includes the QoL of persons living in highly stressful situations, such as migrants and refugees (Hammoudeh *et al*., 2013).

#### The Patient Health Questionnaire (PHQ-9)

The PHQ-9 is a nine-item self-administered scale designed to evaluate depressive symptoms among individuals. The PHQ measures were developed within the PRIME-MD set of instruments and scales and were designed for use in primary care and no psychiatric settings. The nine-item PHQ contains items derived from the DSM-IV classification system pertain to (1) anhedonia, (2) depressed mood, (3) trouble sleeping, (4) feeling tired, (5) change in appetite, (6) guilt or worthlessness, (7) trouble concentrating, (8) feeling slowed down or restless, (9) suicidal thoughts (Gilbody *et al*., 2007).

#### The Beck Hopelessness Scale (BHS)

The BHS is a 20 item self-assessment instrument to measure hopelessness. Respondents are asked to evaluate each of the 20 statements and decide whether they describe their attitude in the previous week (including the assessment day). Nine items are inversely scored to prevent acquiescence. After inversion of the positively worded items, a sum score is calculated. The total score can range from 0 to 20, indicating the number of items endorsed in hopelessness (Kliem *et al*., 2018).

*The General Belongingness Scale (GBS)* was developed by Malone *et al*. (2012) to determine the general belongingness levels of adult individuals. The GBS is a 12-item self-report measure to assess a sense of general of individuals. The GBS evaluates multiple levels of belongingness, including close peer relationships, family, and other community members, beyond interpersonal relationships. The scale is answered on a seven-point Likert-type scale ranging from ‘strongly disagree’ to ‘strongly agree’.

#### Resilience Scale for Adults (RSA)

The RSA is a 33-item self-report scale prepared by Friborg *et al.* (2003) for measuring protective resilience factors among adults. The scale comprises five factors labelled: personal competence (‘I know if I continue, I will succeed’), social competence (‘I can establish friendly relationships easily’), family cohesion (‘Even in difficult situations, my family is optimistic’), social resources (‘There is always someone who helps me when I am in need’), and structured style (‘I sustain my daily rules even in difficult situations’). Respondents rated items using a Likert response format with gradations from 1 (strongly disagree) to 5 (strongly agree).

### Procedures

Data collection was conducted in May 2021 and targeted Palestinians during the war on Gaza and the military violence in the West Bank of Palestine. The sample was recruited online using convenience sampling techniques. The research was conducted in line with the ethical guidelines of the American Psychological Association (APA, 2010) and the Declaration of Helsinki (2013) and had been approved by the An-Najah National University IRB (Protocol number 16 May).

### Data analysis

**Fig. 1.**
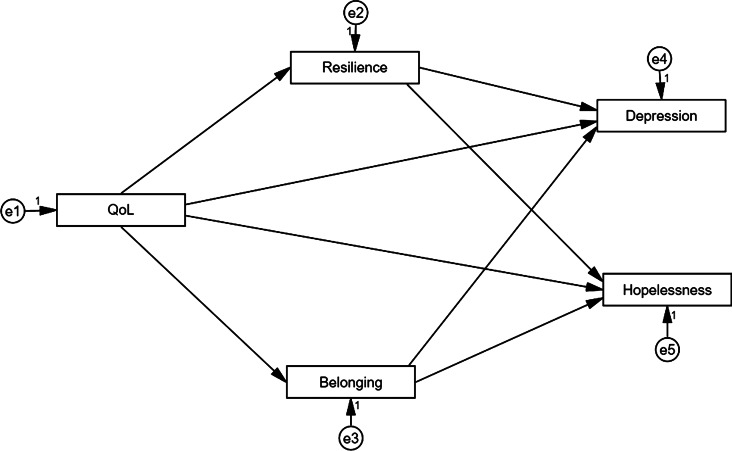
Conceptualized effect for quality of life on depressive symptoms and hopelessness, and the mediating role of resilience and sense of belonging.

Structural equation modeling (SEM) (Gunzler *et al*., 2013) was used to test a conceptual model where resilience and SOB were identified as mediators. Furthermore, the QoL was considered a predictor, and depressive symptoms and hopelessness operated as outcome variables. We explored the statistical distribution of the data for each of the variables. Both kurtosis and skewness values fell inside the recommended cut-offs [–1, +1]. Moreover, we calculated Mahalanobis' distance (*p* < 0.001) for all scores to detect and omit multivariate outliers: no extreme multivariate values were found. We adopted two fit-indexes: absolute and relative. The selected absolute indexes were χ^2^, and normed-χ^2^ (NC) as non-statistically significant χ^2^ value and NC values under 2.0 indicate good fit (Hair *et al*., 2010). Additionally, root mean square error of approximation (RMSEA), normed fit index (NFI), non-normed fit index (NNFI), comparative fit index (CFI), and standardized root mean square. The SEM model (Fig. 1) has been tested using AMOS 25 software for data analysis.

### Findings

Descriptive statistics on QoL, depressive symptoms, hopelessness, SOB, and resilience are given in Table 1. Research participants scored low on depressive symptoms and hopelessness and high scores on QoL, SOB, and resilience. Moreover, scales used in our study showed a high level of reliability (Cronbach's *α*) ranging from 0.831 (*hopelessness*) to 0.932 (*resilience).*

**Table 1.** Descriptive statistics for research variables (*N* = 437)

Variable	Mean	s.d.	Min	Max	Range	Skewness	Kurtosis	Reliability
Quality of life	3.7305	0.49449	2.58	4.71	1.71	−0.227	−0.779	0.867
Depressive symptoms	2.0898	0.75182	1.00	3.88	2.88	0.752	−0.142	0.893
Hopelessness	0.2642	0.11496	0.10	0.55	0.45	0.540	−0.046	0.813
Resilience	4.2003	0.50829	2.58	5.00	2.42	−0.617	0.421	0.932
Belonging	4.1712	0.69416	1.55	5.00	3.45	−1.364	2.172	0.923

Results of the correlational analysis in Table 2 indicated that QoL correlated negatively with depressive symptoms (*r* = −0.603; *p* < 0.01), and hopelessness (*r* = −0.453; *p* < 0.01), and correlated positively with resilience (*r* = 0.534; *p* < 0.05), and SOB (*r* = 0.428; *p* < 0.01). Moreover, depressive symptoms correlated positively with hopelessness (*r* = 0.408; *p* < 0.01), and correlated negatively with resilience (*r* = −0.250; *p* < 0.01), and SOB (*r* = −0.301; *p* < 0.01). Hopelessness correlated negatively with resilience (*r* = −0.478; *p* < 0.01), and SOB (*r* = −0.387; *p* < 0.01). Finally, resilience correlated positively with SOB (*r* = 0.660; *p* < 0.01).

**Table 2.** Correlations among study variables (*N* = 437)

Measures	1	2	3	4	5
1. Quality of life	1	−0.603**	−0.453**	0.534**	0.428**
2. Depressive symptoms		1	0.408**	−0.250**	−0.301**
3. Hopelessness			1	−0.478**	−0.387**
4. Resilience				1	0.660**
5. Belonging					1

### Structural equation model (SEM)

**Fig. 2.**
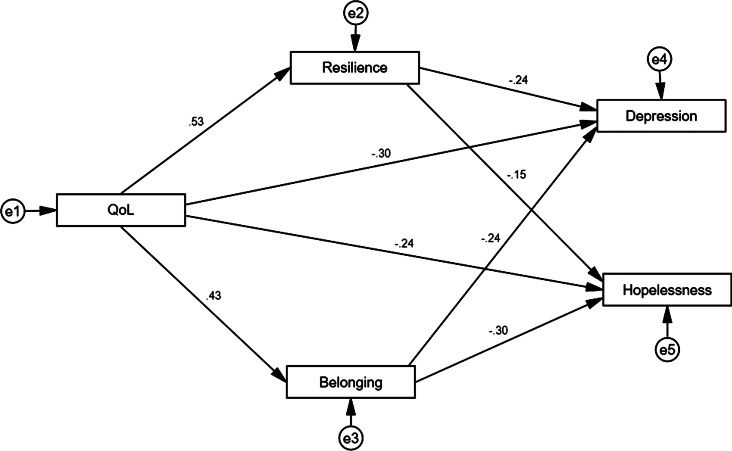
Structural equation modeling for quality of life on depressive symptoms, and hopelessness, and the mediating role of resilience and sense of belonging.

The attained path analysis results are given in Fig. 2. The hypothesized model is in Fig. 1, with QoL as a predictor, SOB and resilience as mediators, and depressive symptoms and helplessness as outcomes were tested across the sample (*n* = 437). Findings of our study indicated that SOB and resilience mediated the correlation between QoL, depressive symptoms, and hopelessness, with a good fit for the data (χ^2^_(4)_ = 221.08; *p* = 0.001; GFI = 0.94; AGFI = 0.93; RMSEA = 0.052; NFI = 0.953; CFI = 0.951).

Regarding (H1), analysis of the moderating effects showed a negative correlation between QoL and depressive symptoms (*β_X, Y_* = 0.603; *p* < 0.001), and hopelessness (*β_X, Y_* = −0.453; *p* < 0.001). Analysis of the path between resilience and depressive symptoms showed a negative effect (*β_M, Y_* = −0.351; *p* < 0.001), while a negative effect in path analysis was also shown between resilience and hopelessness (*β_M, Y_* = −0.145; *p* *<* 0*.001*). Results of path analysis between the SOB and hopelessness showed negative effects (*β_M, Y_* = −0.297; *p* < 0.001), moreover a negative effect was also shown in path analysis between the SOB and depressive symptoms (*β_M, Y_* = −0.242; *p* < 0.001), standardized total direct effect as for (H2).

Finally, concerning the full mediation hypothesis (H3), the model yielded a standardized total effect of resilience on depressive symptoms (*β_X, M_* = −0.236; *p* < 0.001).

However, this effect was composed of a statistically significant indirect effect (via resilience *β_X, M, Y_* = −0.101; *p* *<* 0.05) and a statistically significant direct effect (*β_X, Y, M_* = −0.135; *p* < 0.05). The model also yielded a standardized total effect of self of belonging on depressive symptoms (*β_X, M_* = −0.242; *p* < 0.001). However, this effect was composed of a statistically significant indirect effect (via self of belonging, *β_X, M, Y_* = −0.114; *p* *<* 0.05) and a statistically significant direct effect (*β_X, Y, M_* = −0.128; *p* < 0.01). The model also showed a standardized total effect of self of belonging on hopelessness (*β_X, M_* = −0.236; *p* *<* 0.001). However, this effect was composed of a statistically significant indirect effect (via self of belonging, *β_X, M, Y_* = −0.108; *p* *<* 0.05) and a statistically significant direct effect (*β_X, Y, M_* = −0.128; *p* *<* 0.05). Consequently, the correlation between QoL and depressive symptoms and hopelessness was fully mediated by the SOB and resilience. We also tested the effect of demographic variables in the model (gender, age, and demographic representation), and we found no significant differences due to these variables.

## Discussion

We sought to test the association between QoL, hopelessness, and depressive symptoms and the mediating effect of resilience and SOB in a population undergoing chronic and systematic political violence in oPt. Overall, our hypotheses were confirmed. Hence, we found a direct and inverse association between QoL as a predictor, hopelessness, and SOB as independent variables (H1). Seventy years of military violence and systematic surveillance in almost every sector of Palestinian lives fostered a sense of unsettlement and lack of hope in the population, exposing individuals to psychological burdens, mainly depressive symptoms and a sense of insecurity (Afifi *et al*., 2013). Undermined QoL conditions enforce such a sense of hopelessness. The more all the domains of QoL are compromised by ongoing and widespread violence, the more people are trapped in a pervasive sense of lack of freedom and opportunity, the more they perceive feelings of hopelessness and depression. One possible adverse outcome is the risk of suicidal thoughts and behaviors, reported as a relatively new phenomenon in Palestine (Veronese *et al*., 2021a, 2021b).

On the contrary, individual and collective strategies for survival might control a pervasive lack of hope and depression in the Palestinian civil population. Individually, Palestinians have developed a great extent of resiliency resources that allow individuals to develop creative and powerful forms of resistance in contrasting hopelessness and depressive symptoms (Ryan, 2015; Keelan and Browne, 2020). The Palestinian endurance to ongoing hardships is a means of resistance to an intractable conflict worsening over the years and a protective factor against depression despite the compromised living conditions (Nguyen-Gillham *et al*., 2008; Aitcheson *et al*., 2017). Moreover, our findings showed that belonging is an essential collective factor contributing to mitigating the lack of hope and depression. In fact, the collectivistic nature of the Palestinian society confirms a sense of commonality and being part of an oppressed indigenous population is an effective protective factor against the burdens of the occupation (Afana *et al*., 2020). Belonging means a source for resisting the sense of loss and dispossession that is characterizing the Palestinian collective trauma (Beauregard *et al*., 2017); thus, being collectively part of the Palestinian struggle for existence fuels hope and reduce the risk of feeling broken and depressed (Barber *et al*., 2016).

Our findings indicate how QoL might be a core determinant of individuals' psychological suffering. Such a crucial role of QoL is confirmed by the political and environmental turbulences that characterize Palestinian lives (Barber *et al*., 2014). Thus, Palestinians might contrast such disrupted living conditions via individual (resilience) and collective (SOB) resources. These findings might indicate that sole resilience can be insufficient to provide people with adequate functioning sources. At the same time, a SOB and resiliency together will contribute to controlling hopelessness and symptoms of depression due to precarious QoL. In other words, despite a diffuse compromised QoL, Palestinians can conserve good psychological functioning in terms of hope and lessened depressive symptoms when they can access individual sources of resilience and a sense of community and belonging.

Our study sheds light on resilience and SOB as protective factors against ongoing traumatic experiences among Palestinians (Marie *et al*., 2018). However, severely compromised QoL could neutralize such competencies, re-enhancing feelings of desperation and hopelessness (Hobfoll *et al*., 2011). Years of political conflict are disrupting individual and collective survival skills, exposing people and community to psychological burdens and creating personal suffering and internal divisions.

Our findings can be considered for some practical implications. On the one hand, clinical interventions that do not consider the attempt to improve the QoL among the oppressed risk being ineffective and incapable of mobilizing natural resources and skills of resistance that can protect people's mental well-being. Moreover, potentiating personal resilience and a SOB among individuals affected by chronic violence and severe human rights violations might help people conserve proper psychological functioning even when their QoL is compromised. Psychosocial support must be oriented to improve people's life opportunities and potentiating their individual and collective resources against loss of hope and depressive symptoms.

## Conclusions

People affected by chronic and systematic violence are commonly compromised in their mental health. Such individuals lack personal and collective resources to confront life hardships physically and psychologically. Most often, those people have lost their hope and deal with a deep sense of desperation. The most direct consequences are the loss of resources to cope with difficulties, reproducing cycles of violence and reducing the SOB to a victimized and passivized group (Nguyen-Gillham *et al*., 2008). Our study showed that Palestinian people in maintaining individual competencies and a collective SOB in an environment characterized by a disrupted QoL. Future research should be addressed to understand better the features of resilience and SOB that can help maintain psychological functioning in conditions of chronic and ongoing violence, the personal and historical antecedents of such protective factors and the factors that can directly or indirectly undermine them.

Before closing, some caveat in our study is worthy of being discussed and addressed. The cross-sectional nature of the study will prevent us from generalizing our results and drive causalistic conclusions. However, the association between the study variables provides promising arguments that might be tested in future longitudinal or experimental research designs. The online recruitment of the sample could have limited the access to the research of the most unwell groups, providing more insight into how resilience and SOB worked in regulating the association between QoL, hopelessness, and depression in more disadvantaged strips of the Palestinian population. In the future, more traditional pencil and paper administration of the questionnaires will shed light on the tested conceptual model in the most deprived people in Palestine and elsewhere. However, we must acknowledge that the Palestinian population dwells in significantly compromised living conditions, collectively experiencing hopelessness and desperation. Thus, our work focused on addressing diversity if we consider Palestinians as an oppressed indigenous minority group struggling for its psychological and physical survival and the recognition of its existence as a national and ethnic group.

## Data

All data generated or analyzed during this study are included in this published article.
